# Quality and accuracy of radiomics models in predicting KRAS status in lung cancer: a systematic review and meta-analysis

**DOI:** 10.3389/fonc.2025.1701122

**Published:** 2026-01-09

**Authors:** Xindong Luo, Ziqiang Wang, Di Lu, Yaping Wang, Wenliang Wang, Pengcheng Dong, Yunjiu Gou, Yayuan Yang

**Affiliations:** 1 Key Laboratory of Veterinary Pharmaceutical Development, Ministry of Agricultural and Rural Affairs, Lanzhou Institute of Husbandry and Pharmaceutical Sciences of Chinese Academy of Agricultural Sciences, Lanzhou, China; 2 The First Clinical Medical College of Gansu University of Chinese Medicine, Gansu Provincial Hospital, Lanzhou, China; 3 Department of Thoracic Surgery, Gansu Province Hospital, Lanzhou, China

**Keywords:** KRAS gene mutation, lung cancer, cancer, radiomics, non-small cell lung cancer, deep learning

## Abstract

**Introduction:**

This study aimed to systematically evaluate the diagnostic performance of radiomics-based models in predicting KRAS gene mutations in lung cancer and quantitatively analyze the methodological quality and reporting standardization of related studies.

**Methods:**

Original studies evaluating radiomics models for predicting KRAS mutation status in lung cancer patients were identified through systematic searches of databases including PubMed, Embase, China National Knowledge Infrastructure (CNKI), Web of Science, and the Cochrane Library (from inception to June 2025). The Quality Assessment of Diagnostic Accuracy Studies-2 (QUADAS-2) tool was used to assess diagnostic bias risk, the Radiomics Quality Score (RQS, comprising 16 items with a total score of 36) was employed to quantify methodological quality, and the METRICS (10 criteria, 100-point scale) was applied to evaluate reporting standardization. A single-arm meta-analysis was conducted on 20 eligible studies (total sample size: 4,953 cases) to calculate pooled sensitivity, specificity, and the area under the summary receiver operating characteristic curve (SROC AUC). External validation was performed using validation cohorts from 12 studies.

**Results:**

The mean RQS score of included studies was 9.86 ± 3.7 (range: 4–15, representing 27.4% ± 10.3% of the maximum score), with a mean METRICS score of 59.95 ± 13.5%. The primary analysis revealed pooled sensitivity of 0.80 (95% CI: 0.76–0.83), specificity of 0.78 (95% CI: 0.75–0.82), and AUC of 0.85 (95% CI: 0.82–0.88). Validation cohort results were consistent: sensitivity 0.79 (95% CI: 0.73–0.84), specificity 0.77 (95% CI: 0.71–0.82), and AUC 0.85 (95% CI: 0.81–0.88). Significant heterogeneity was observed among studies, but meta-regression and subgroup analyses (based on key methodological variables such as modeling algorithms, imaging modalities, RQS scores, and validation methods) confirmed stable results across subgroups, demonstrating clinical applicability.

**Conclusion:**

Radiomics models exhibit moderate diagnostic performance in predicting KRAS mutations in lung cancer. Future efforts should strictly adhere to relevant guidelines, strengthen model validation, and standardize workflows to enhance the practical value of radiomics in precision oncology.

**Systematic review registration:**

https://www.crd.york.ac.uk/PROSPERO/view/CRD420251148699, identifier CRD420251148699.

## Introduction

Lung cancer, the malignancy with the highest cancer-related mortality, accounts for approximately 1.8 million deaths annually, representing 18.4% of all cancer-related fatalities. Among these cases, non-small cell lung cancer (NSCLC) constitutes 85% of all lung cancer diagnoses, with approximately 30% of NSCLC patients presenting at advanced stages (III or IV) upon initial diagnosis (1–3). For patients ineligible for surgical intervention, targeted therapy has emerged as the standard treatment modality (4, 5). The progressive development and clinical implementation of driver gene-based targeted therapies have revolutionized therapeutic paradigms for lung cancer patients (6–8).

Sotorasib and Adagrasib have demonstrated efficacy in prolonging survival and improving safety profiles among patients with KRAS-mutated lung cancer. Furthermore, following treatment with KRAS inhibitors, the tumor immune microenvironment transiently shifts to a less immunosuppressive state, exhibiting heightened sensitivity to therapies that promote antitumor immune responses. This observation underscores the potential for combining KRAS inhibitors with immunotherapy to achieve greater clinical benefits (9, 10).

Traditional KRAS mutation detection relies on needle biopsy, which is associated with limitations such as sampling bias, high costs, prolonged processing times, and inaccurate results due to difficulties in obtaining adequate tissue samples (11). Radiomics, a high-throughput approach, transforms medical imaging into mineable data (12, 13), encompassing both quantitative and visual features (14, 15). Its underlying hypothesis posits that tumor microstructural changes and heterogeneity can be reflected through imaging (16), thereby offering potential for predicting KRAS status and enabling personalized treatment strategies.

In recent years, research on radiomics-based prediction of KRAS mutations in lung cancer has surged. Given the critical importance of developing reliable predictive tools for clinical diagnosis and treatment, there is an urgent need to systematically summarize the predictive performance and clinical translational potential of radiomics models. Therefore, this systematic review and meta-analysis aim to clarify the value of radiomics in predicting KRAS mutation status in lung cancer.

## Methods

The Preferred Reporting Items for a Systematic Review and meta-analysis of Diagnostic Test Accuracy Studies (PRISMA-DTA) were used to guide the research. The research has been registered to the PROSPERO database (CRD420251148699) for systematic reviews.

### Literature retrieving

We conducted a computerized search across PubMed, Scopus, Embase, CNKI, Web of Science, and Cochrane Library databases to identify original studies that employed imaging radiomics analysis for predicting KRAS mutation status in lung cancer. The search strategy was formulated in accordance with the PICO (Population, Intervention, Comparison, Outcome) principle to enable sensitive screening of relevant studies. The search terms included “lung cancer or non-small cell lung cancer,” “radiology or radiomics,” and “KRAS.” All clinical original studies reported and published up to June 2025 were included. Detailed search criteria are provided in **Supplementary Table 1**.

### Study inclusion

After removing duplicate references, studies meeting the following criteria were selected: (1) Patients with lung cancer diagnosed by pathological examination; (2) Prediction of KRAS status using artificial intelligence algorithms based on preoperative imaging data, namely positron emission tomography/computed tomography (PET/CT), computed tomography (CT), and magnetic resonance imaging (MRI); (3) Any study design, including retrospective and prospective observational studies. The following criteria were employed to exclude relevant studies: (1) Duplicate studies using the same patients and data; (2) Case reports, review articles, letters, conference reports, and editorials; (3) Studies not relevant to the topic. Initially, the titles and abstracts of all retrieved papers were reviewed, followed by a full-text review of potentially eligible papers. A double-blind screening mechanism was adopted: two reviewers (XDL, ZQW) with four years of experience in lung radiomics analysis independently conducted the initial screening of titles/abstracts and subsequent full-text screening. Disagreements were resolved through arbitration by a third party (YJG, a senior researcher with fifteen years of experience in lung cancer diagnosis and treatment as well as meta-analysis). In cases of missing data, the corresponding authors were contacted via email to obtain the original data; studies were excluded if no response was received after three attempts.

### Data extraction

The following data were extracted from the included studies: (1) Study characteristics: author (publication year), country of the institution to which the corresponding author belongs, and type of study design; (2) Patient sample size; (3) Characteristics of radiomics model construction, including imaging modality, image segmentation method, feature extraction method, and modeling algorithm; (4) Diagnostic performance metrics of artificial intelligence models: area under the curve (AUC), sensitivity, and specificity.

If a study reported accuracy data for multiple models, we selected the 2×2 contingency table corresponding to the model with a medium Youden’s index. If the study provided both internal and external validation results, only the 2×2 contingency table corresponding to the external validation accuracy data was extracted. When necessary, **Supplementary Files** of the included studies were also screened to obtain the required data.

### Quality assessment

Three tools were employed to evaluate the quality of included studies:(1) Quality Assessment of Diagnostic Accuracy Studies-2 (QUADAS-2): This tool assessed the risk of bias across four domains: patient selection, index test, reference standard, and flow and timing.

(2) Radiomics Quality Score (RQS) Criteria: Comprising 16 dimensions (e.g., imaging acquisition, feature extraction, modeling methodology, model validation, clinical applicability), this scoring system assigns a total possible score of 36 points. Based on international consensus, studies with an RQS ≤ 5 are classified as “very low quality,” while those with an RQS ≥ 9 are deemed “adequate quality” (17). (3) Methodological Radiomics Score (METRICS): A structured assessment tool developed through international consensus, METRICS employs a hierarchical classification framework to grade methodological variations and allocate weights accordingly.

### Operational procedures

Data extraction and quality assessment adhered to a standardized protocol:

Training Phase: Three authors (XDL, ZQW, JLZ) independently reviewed three included studies to reach consensus on interpreting data extraction and quality assessment forms, with final definitions confirmed by the senior author (YJG).Independent Execution: Two authors (XDL, ZQW) independently performed data extraction and quality assessment tasks, with METRICS scores facilitated by a user-friendly online tool (https://metricsscore.github.io/metrics/METRICS.html).Data Integration: Consensus-confirmed information from both reviewers was consolidated into a final electronic data spreadsheet.

### Statistical analysis

The κ statistic quantified inter-reviewer agreement during full-text manuscript evaluations. Agreement levels were categorized into six grades based on κ values: almost perfect (0.81–1.0), substantial (0.61–0.80), moderate (0.41–0.60), fair (0.21–0.40), slight (0.0–0.20), and poor (<0.0) (18).

#### Data processing

Training and validation datasets were independently extracted from relevant studies. Extracted data (sample size, sensitivity, specificity) were input into Review Manager 5.4 (https://community.cochrane.org/revman) to calculate true positives, true negatives, false positives, and false negatives. Paired forest plots displaying sensitivity, specificity, and their 95% confidence intervals (CIs) were generated using the “midas” command in Stata 17.0. Receiver operating characteristic (ROC) curves were plotted with sensitivity on the x-axis and specificity on the y-axis. Additionally, the Pearson correlation coefficient was used to examine the relationship between RQS and METRICS scores.

#### Heterogeneity assessment

Initial heterogeneity evaluation involved visual inspection of ROC plots and forest plots. For precise quantification, the I² statistic was employed—values >75% indicated substantial heterogeneity, with bilateral P < 0.05 as the threshold for statistical significance. Meta-regression and subgroup analyses were conducted to explore associations between methodological factors and inter-study heterogeneity.

#### Publication bias evaluation

With >10 studies included, publication bias was assessed. Initial evaluation involved visual inspection of funnel plot asymmetry (generated by plotting effect size against precision), followed by formal assessment using Deeks’ test, with the diagnostic odds ratio (DOR) serving as the accuracy metric.

## Results

### Study selection

**Figure 1:**
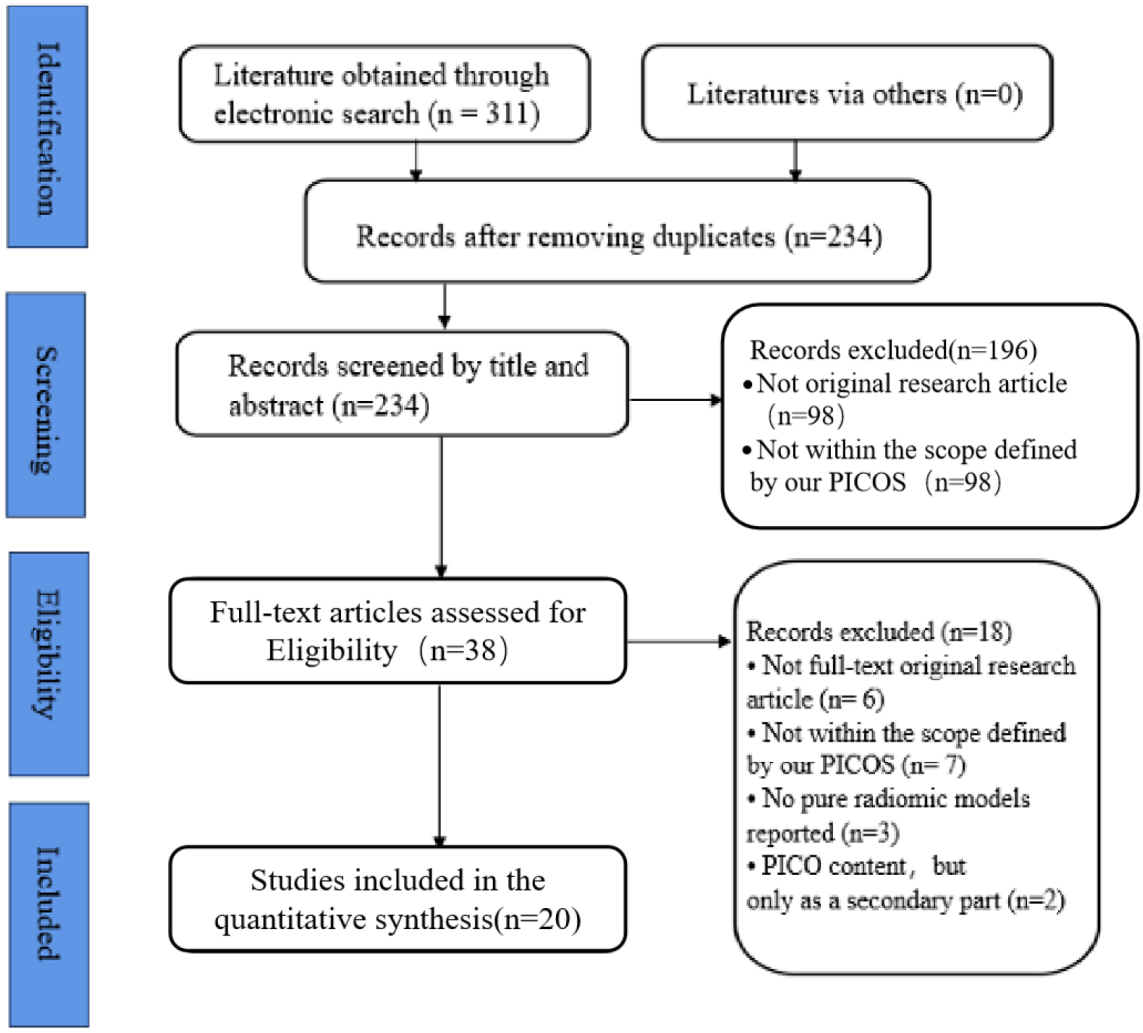
Flow diagram for study inclusion.

We conducted searches across the PubMed, Scopus, Embase, CNKI, and Web of Science databases, identifying a total of 437 relevant articles. Subsequently, 203 duplicate articles were removed, leaving 234 studies. After screening the titles and abstracts of these 234 studies, 197 articles that did not meet the preset criteria regarding language, study type, and PICO (Population, Intervention, Comparison, Outcome) standards for this study were excluded. Following full-text review, an additional 18 articles were excluded, ultimately resulting in the inclusion of 20 articles (**Figure 1**).

### Study characteristics

The summarized information of the 20 included studies is presented in **Table 1**, encompassing a total of 4,953 patients. The majority of the literature originated from China (12 articles, accounting for 60%). The research time span extended from January 2014 to June 2025, with the number of published articles showing an increasing trend year by year, reaching a peak between 2023 and 2025 (10 studies). All studies employed a retrospective design. Among them, 12 studies divided their cohorts into training and validation sets, while the remaining studies only established training sets. In terms of imaging examinations, 13 studies utilized CT-based radiomics analysis, 6 were based on PET/CT, and 1 on MRI imaging. Fifteen studies adopted three-dimensional (3D) segmentation. Regarding model algorithms, 5 studies constructed models using deep learning algorithms, 8 studies established diagnostic models by integrating clinical parameters, and 7 studies conducted modeling based on traditional radiomics methods. Cohen’s κ was used to evaluate the agreement between the two raters throughout the review process, and the results demonstrated good agreement (κ = 0.87).

**Table 1:** Basic characteristics of included studies.

Study(publication year)	Location	Sample size	ROI	Imaging modality	Feature extraction software	Segmentation	Features type	Deep learning	Validation	Clinical factors
Rizzo (2014) (19)	Italy	285	NR	CT	NR	manual	NR	NO	NO	NO
Velazquez (2018) (20)	China	763	NR	CT	MATLAB	manual	Morphological	NO	NO	YES
Rizzo (2019) (21)	Italy	122	NR	CT	Pyradiomics	manual	first-order,shape,GLCM,GLDM,GLRLM,GLSZM,NGTDM	NO	NO	NO
Dong (2020) (22)	China	363	3D	CT	NR	manual	First-order, second-order and higher-order	YES	YES	NO
Moreno (2021) (23)	Colombia	99	3D	CT	NR	NR	shape-based, first order GLCM, GLDM,GLRLM,GLZLM,NGTDM	YES	NO	NR
Nguyen (2021) (24)	China	161	NR	CT	PyRadiomics	manual	first-order, shape, GLCM, GLDM,GLRLM, GLSZM, NGTDM	NO	YES	NO
Zhang (2021) (25)	China	134	3D	PET/CT	Pyradiomics	Semi-automatic	first-order,shape,GLCM,GLDM,GLRLM,GLSZM,NGTDM	NO	NO	YES
Shiri (2022) (26)	Switzerland	168	3D	PET/CT	MATLAB	manual	first-order, GLCM,LRLM,GLSZM,GLDZM,NGTDM	NO	YES	NO
Wang (2022) (27)	China	258	3D	PET/CT	Pyradiomics	manual	first-order,shape,GLCM,GLDM,GLRLM, GLSZM, NGTDM	NO	YES	NO
Chen (2023) (28)	China	246	3D	CT	pyradiomics	Semi-automatic	First-order, second-order and higher-order	NO	YES	YES
Prencipe (2023) (29)	Italy	55	NR	CT	PyRadiomics	NR	GLCM,GLSZM,GLRLM,NGTDM, GLDM	NO	YES	YES
Zhang (2023) (30)	Germany	119	3D	PET/CT	Pyradiomics	Semi-automatic	shape-based, first order GLCM, GLDM,GLRLM,GLZLM,NGTDM	YES	YES	NO
Kohan (2024) (31)	Canada	157	3D	CT	LIFEx	Semi-automatic	NR	NO	NO	YES
Li (2024) (32)	China	120	^3D^	PET/CT	Pyradiomics	manual	first-order, shape, GLDM,GLRLM, GLSZM,	NO	NO	YES
Lv (2024) (33)	China	317	3D	MRI	3D Slicer	manual	first-order features, shape-based features, GLCM, GLRLM, GLSZM,GLDM, NGTDM.	NO	YES	NO
Xue (2024) (34)	China	124	3D	CT	NR	Semi-automatic	NR	YES	YES	NO
Xu (2024) (35)	China	366	3D	PET/CT	Pyradiomics	Semi-automatic	first-order features, shape-based features, GLCM, GLRLM, GLSZM,GLDM, NGTDM.	NO	YES	YES
Fu (2025) (36)	China	69	3D	CT	Artificial Intelligence Kit	manual	NR	NO	NO	NO
Mahmoud (2025) (37)	China	815	3D	CT	Pyradiomics	automatically	first-order features, shape-based features, GLCM, GLRLM, GLSZM,GLDM, NGTDM.	YES	YES	YES
Schöneck (2025) (38)	Germany	212	3D	CT	PyRadiomics	manual	first-order, shape, GLCM, GLDM, GLRLM, GLSZM, NGTDM	NO	YES	NO

NR, Not Reported;GLCM:Gray-Level Co-occurrence Matrix;GLDM:Gray-Level Dependence Matrix;GLRLM:Gray-Level Run-Length Matrix;GLSZM:Gray-Level Size Zone Matrix;NGTDM:Neighborhood Gray-Tone Difference Matrix.

### Quality assessment

**Figure 2:**
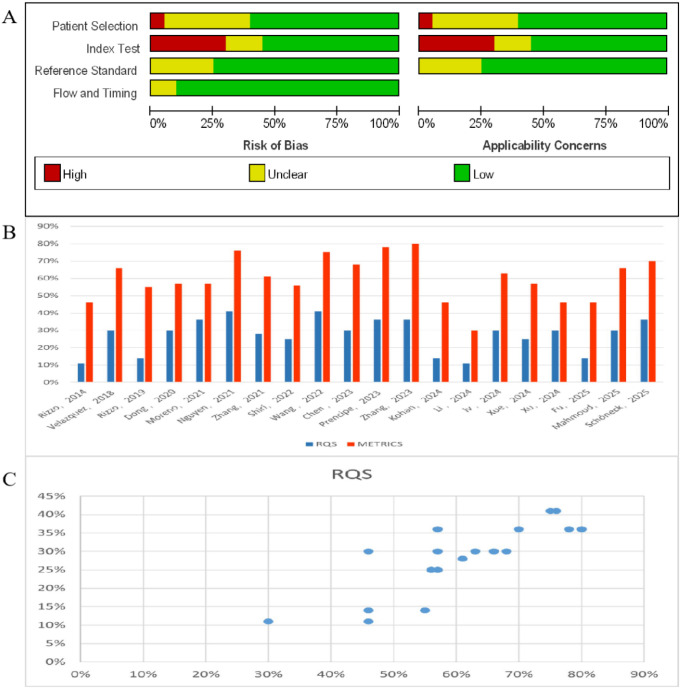
Quality assessment of included articles. **(A)** The bias risk assessment of included studies utilizing the QUADAS-2 scale. **(B)** RQS and METRICS scores of included articles. **(C)** Relationship between METRICS and RQS for each included study, illustrated by scatter plot.

The quality of the included studies was assessed using the QUADAS-2 tool, with the results presented in **Figure 2A**. Overall, the methodological quality of the selected studies was moderate, with a prevalent risk of bias primarily stemming from the patient selection process. This was manifested in the lack of transparency in inclusion/exclusion criteria (e.g., failure to specify whether all consecutive patients were included), the absence of clear criteria for setting decision thresholds in diagnostic models, and the failure to validate established models in independent datasets. The latter two factors—lack of model validation criteria and lack of external validation—were critical issues affecting the applicability of the studies.

A summary of study quality based on the Radiomics Quality Score (RQS, with a maximum score of 36) and the Methodological Radiomics Score (METRICS) is shown in **Figures 2B** and **C**. The average total RQS score across all 20 studies was 9.86 ± 3.7 (equivalent to 27.4% ± 10.3% of the maximum score). A significant positive correlation was observed between METRICS scores and RQS scores (Pearson correlation coefficient r = 0.836, p < 0.0001, **Figure 2C**), with an average METRICS score of 59.95 ± 13.5%. In terms of specific quality dimensions, most studies met the criteria for implementing model validation, disclosing radiomics features, evaluating discrimination/calibration, reporting imaging acquisition strategies, and integrating non-radiomics clinical variables. According to the METRICS assessment criteria, the quality grades of the 20 studies were distributed as follows: excellent (3 studies), good (11 studies), fair (5 studies), and poor (1 study).

### Data analysis

**Figure 3:**
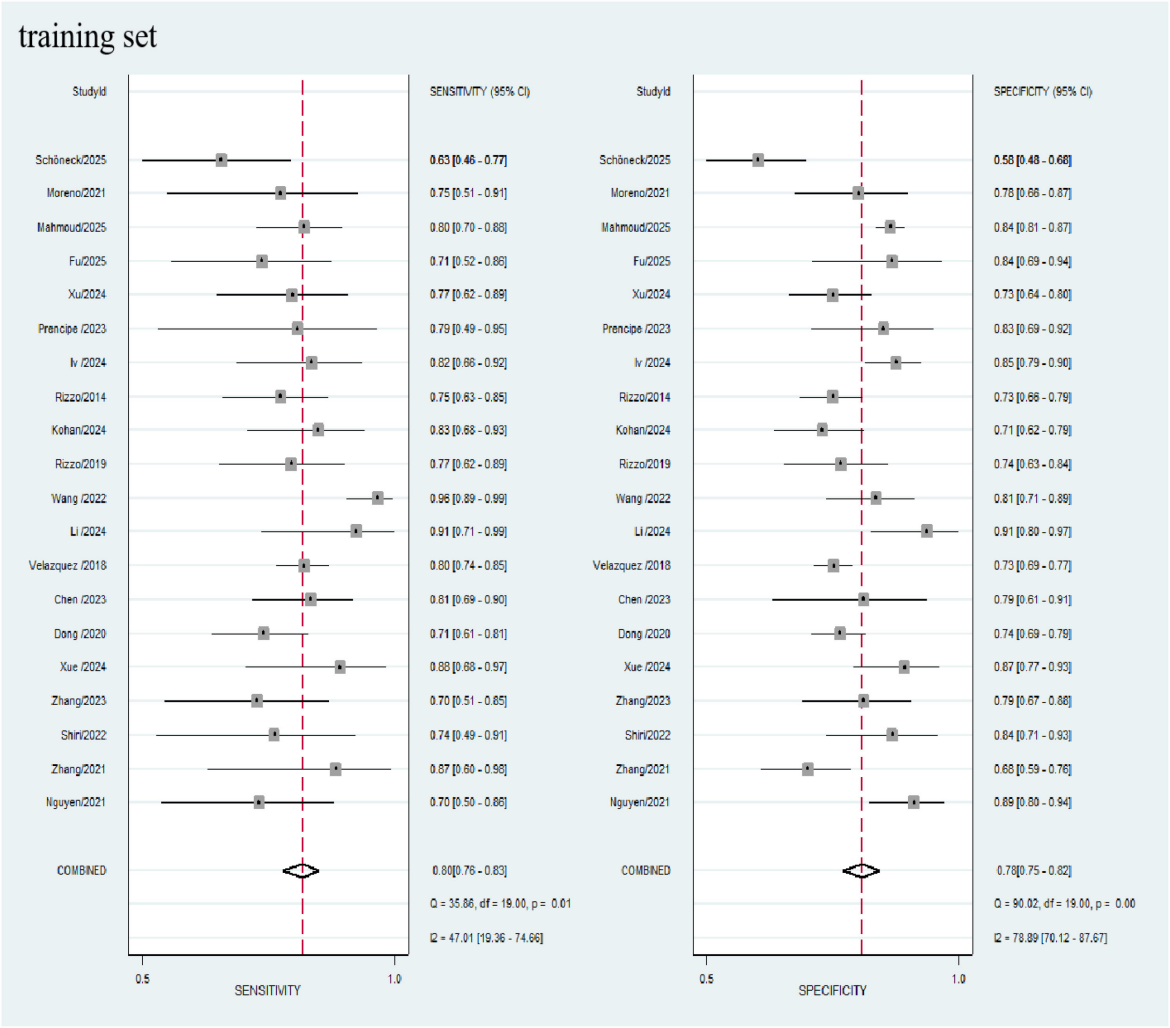
Coupled forest plots of pooled sensitivity and specificity of diagnostic performance of predicting KRAS mutations in lung cancer patients for training cohort. The numbers are pooled estimates with 95% CIs in parentheses; horizontal lines indicate 95% CIs.

**Figure 4:**
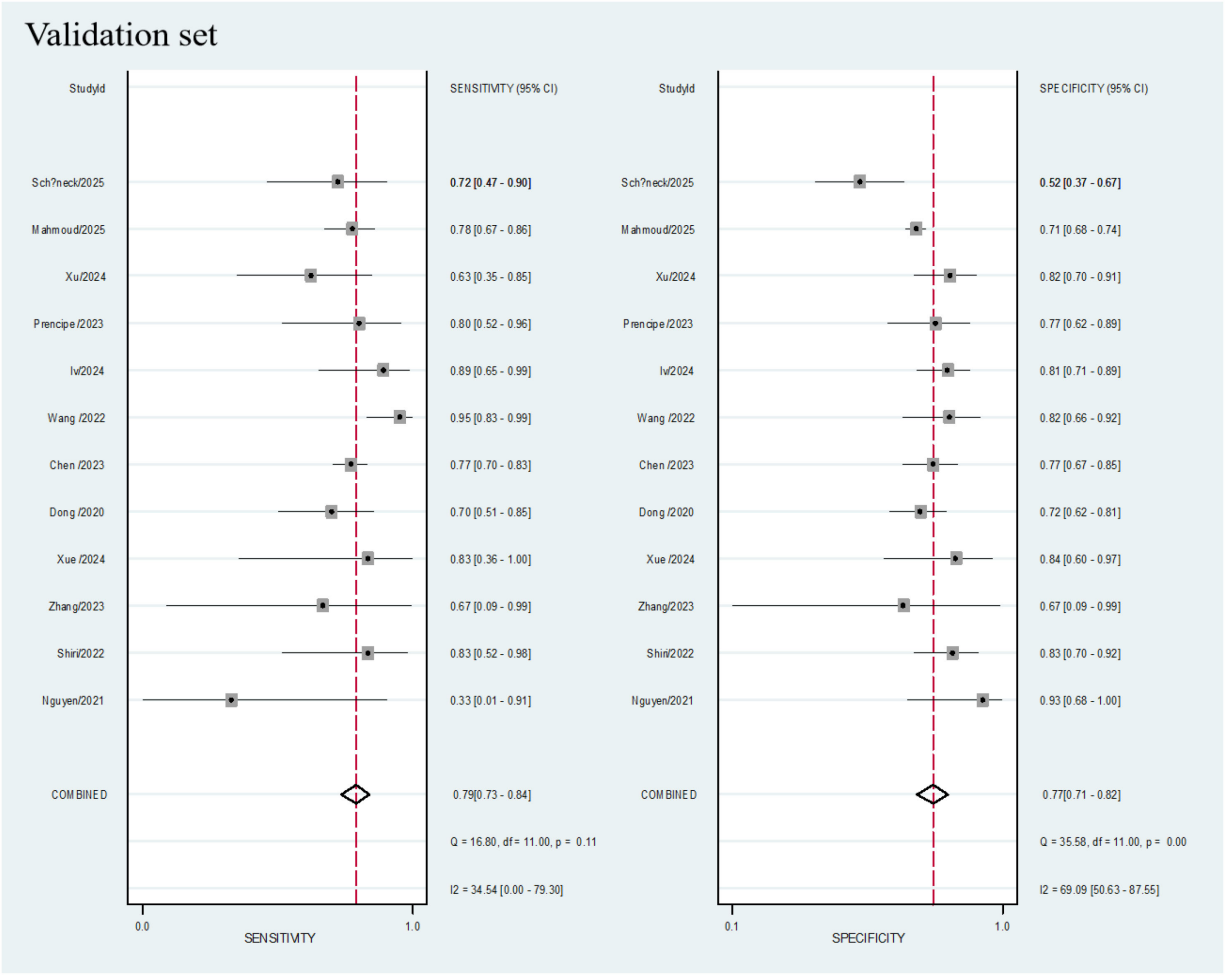
Coupled forest plots of pooled sensitivity and specificity of diagnostic performance of predicting KRAS mutations in lung cancer patients for validation cohort. The numbers are pooled estimates with 95% CIs in parentheses; horizontal lines indicate 95% CIs.

**Figure 5:**
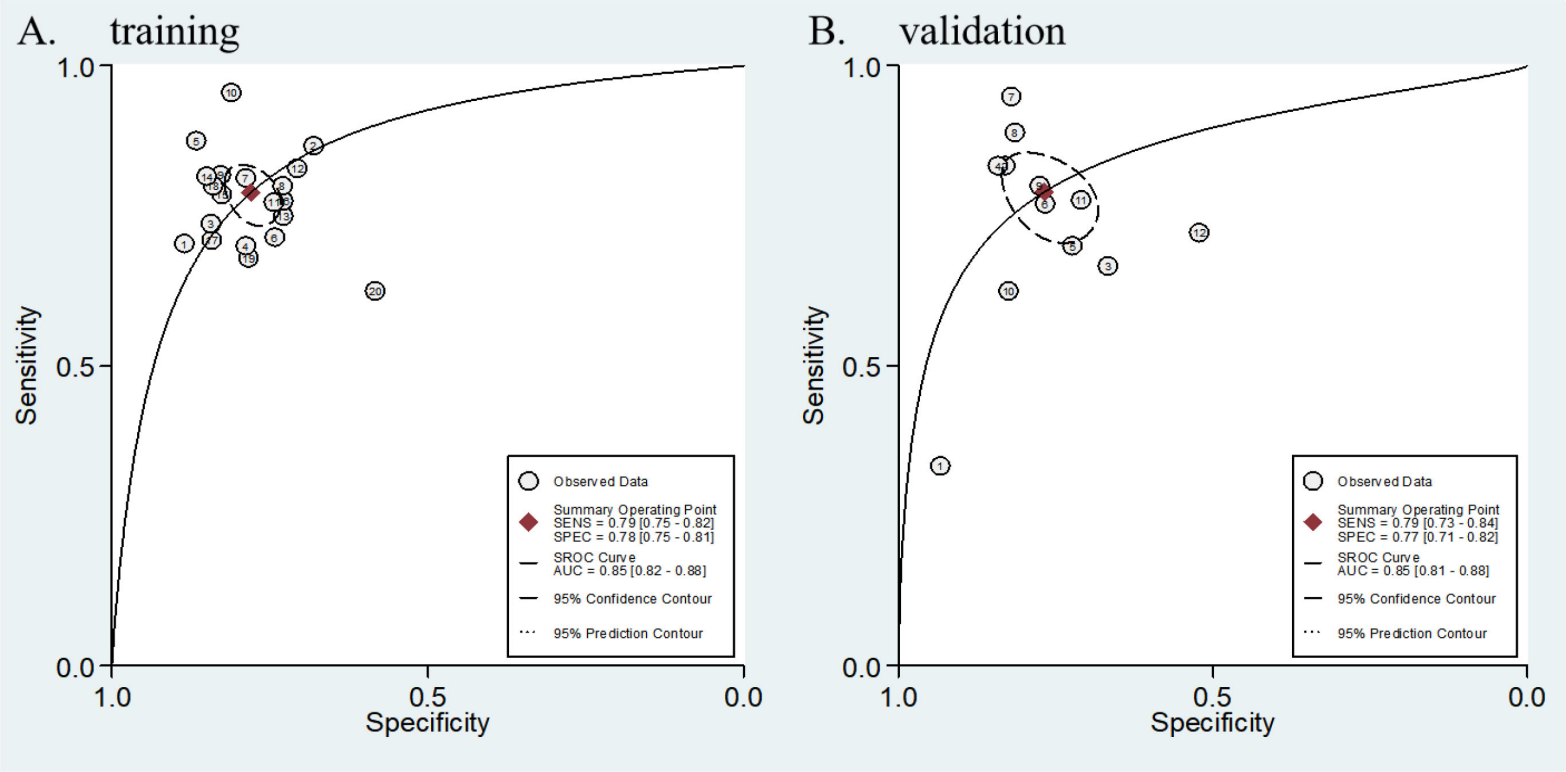
Coupled SROC curves for diagnostic performance of AI in predicting KRAS mutations in lung cancer patients: **(A)** training cohort SROC curve (95% CI: solid line; 95% prediction region: shaded area) and **(B)** validation cohort SROC curve. Significant discrepancy between 95% confidence intervals and prediction regions indicates substantial heterogeneity across studies.

A total of 20 studies were included in the meta-analysis. In the training cohorts of all the studies, the pooled sensitivity, specificity, positive likelihood ratio (PLR), negative likelihood ratio (NLR), and diagnostic odds ratio (DOR) of radiomics for evaluating the KRAS status in lung cancer patients were 0.80 (95% CI: 0.76 - 0.83), 0.78 (95% CI: 0.75 - 0.82), 3.6 (95% CI: 3.0 - 4.3), 0.27 (95% CI: 0.22 - 0.33), and 13 (95% CI: 9 - 19), respectively. Among the 12 validation cohorts, the pooled sensitivity, specificity, PLR, NLR, and DOR were 0.79 (95% CI: 0.73 - 0.84), 0.77 (95% CI: 0.71 - 0.82), 3.4 (95% CI: 2.6 - 4.4), 0.27 (95% CI: 0.21 - 0.36), and 12 (95% CI: 7 - 20), respectively (see the **Table 2**). Forest plots for the training and validation cohorts are presented in **Figures 3** and **4**, respectively. As shown in **Figures 5A** and **B**, the areas under the curve (AUCs) for the training and validation cohorts were 0.85 (95% CI: 0.82 - 0.88) and 0.85 (95% CI: 0.81 - 0.88), respectively.

**Table 2:** Results of meta-analysis.

Dataset type	Sensitivity	Specificity	Positive likelihood ratio	Negative likelihood ratio	Diagnostic odds ratio	AUC
Training Set	0.80 (95%CI: 0.76-0.83)	0.78 (95%CI: 0.75-0.82)	3.6 (95%CI:3.0-4.3)	0.27 (95%CI:0.22-0.33)	13 (95%CI:9-19)	0.85 (95%0.82-0.88)
Validation Set	0.79 (95%CI: 0.73-0.84)	0.77 (95%CI: 0.71-0.82)	3.4 (95%CI:2.6-4.4)	0.27 (95%CI:0.21-0.36)	12 (95%CI:7-20)	0.85 (95%CI0.81-0.88)

### Meta-regression and subgroup analysis

To identify sources of heterogeneity, we conducted meta-regression and subgroup analyses, taking into account four variables: the inclusion of clinical parameters, the Radiomics Quality Score (RQS), imaging modality, validation method (internal cross-validation versus external validation), and the application of deep learning methods. The meta-regression results indicated that the use of different imaging modalities (*P* = 0.032), the application of deep learning algorithms (*P* = 0.014), RQS scores (*P* = 0.021), and differences in validation methods (*P* = 0.028) might be factors contributing to significant variations in diagnostic performance among studies and generating heterogeneity. However, the inclusion of clinical features in model construction did not appear to be a source of heterogeneity (*P* = 0.63). Detailed results of the subgroup analyses are presented in **Table 3**.

**Table 3:** The pooled results from subgroup analysis.

Subgroups	Main effect	No. of datasets	AUC	Sensitivity (95%CI)	Specificity (95%CI)	DOR (95% CI)	I^2^%	P (P value)
RQS得分								0.021
	≥9	15	0.85	0.78 (0.73-0.87)	0.76 (0.72-0.83)	14 (6-30)	48.11	
	≤5	5	0.90	0.84 (0.77-0.92)	0.86 (0.80-0.91)	16 (10-31)	37.1	
Imaging modality								0.032
	CT	13	0.85	0.80 (0.76-0.85)	0.81 (0.75-0.84)	17 (8-43)	46.2	
	PET/CT	6	0.91	0.84 (0.76-0.91)	0.86 (0.81-0.91)	33 (14-74)	36.1	
	MRI	1	–	–	–	–	–	-
Deep learning								0.014
	YES	5	0.88	0.83 (0.78-0.86)	0.82 (0.78-0.88)	23 (**9**-53)	45.0	
	NO	15	0.84	0.79 (0.74-0.87)	0.79 (0.75-0.86)	14 (6-29)	57.8	
Validation								0.028
	external validation	8	0.84	0.78 (0.72-0.86)	0.79 (0.71-0.86)	12 (4-23)	39.2	
	internal cross-validation	4	0.91	0.84 (0.78-0.90)	0.84 (0.77-0.90)	16 (7-31)	67.3	
Clinical parameter								0.63
	YES	8	0.85	0.79 (0.72-0.86)	0.78 (0.73-0.85)	14 (6-27)	33.1	
	NO	12	0.86	0.80 (0.78-0.85)	0.79 (0.77-0.85)	17 (8-35)	49.2	

There was no significant difference in the diagnostic performance of the models, regardless of whether clinical parameters were included, with corresponding areas under the curve (AUCs) of 0.85 and 0.86, respectively. Among the six studies that constructed radiomics models using PET/CT image features, the diagnostic performance was the best (AUC = 0.91), while the AUC for models based on CT images was 0.85. Both the PET/CT subgroup and the CT subgroup exhibited low heterogeneity, at 36.1% and 46.2%, respectively.

The diagnostic performance of the subgroup using deep learning algorithms was superior to that of the subgroup using traditional machine learning algorithms (AUC 0.88 versus 0.84), and the deep learning subgroup had lower heterogeneity (I² = 45.0%), while the traditional algorithm subgroup had moderate heterogeneity (I² = 57.8%). Meta-regression using RQS as a continuous covariate did not reveal an association with the pooled results. Re-analysis based on RQS classification (very low ≤ 5 versus appropriate ≥ 9) showed that the low RQS subgroup had better diagnostic performance (AUC = 0.90 versus 0.85), and both the low RQS subgroup and the appropriate RQS subgroup had low heterogeneity, at 37.1% and 48.11%, respectively.

**Figure 6:**
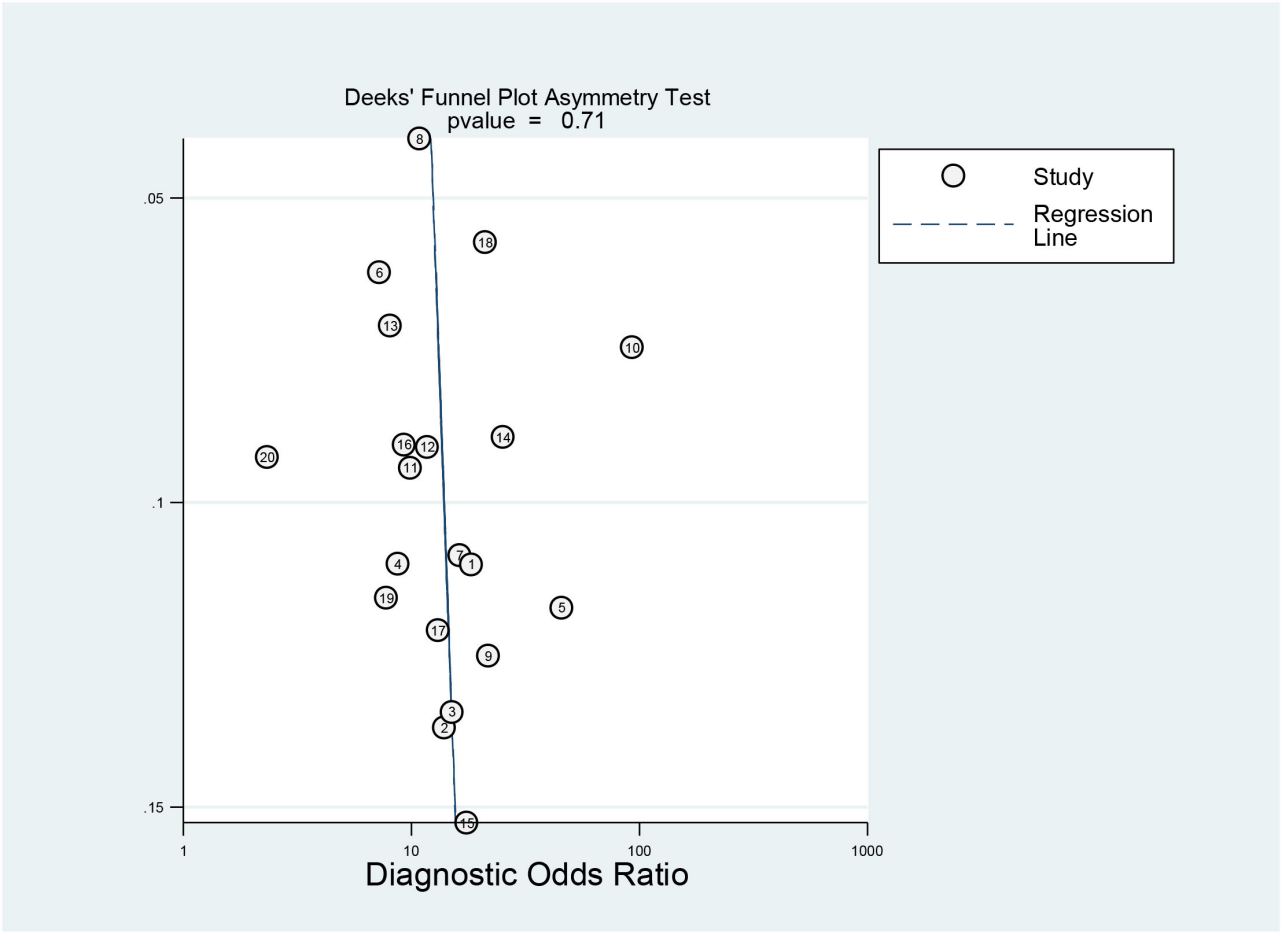
Effective sample size (ESS) funnel plots and the associated regression test of asymmetry, as reported by Deeks et al. A p value < 0.10 was considered evidence of asymmetry and potential publication bias.

The diagnostic efficacy of the internal cross-validation group was superior to that of the independent external validation group. The internal cross-validation group had moderate heterogeneity (I² = 67.3%), while the external validation group had low heterogeneity (I² = 39.2%). The P-value for Deeks’ funnel plot asymmetry test was 0.71, indicating no significant evidence of publication bias among the included studies (**Figure 6**).

## Discussion

To the best of our knowledge, this is the first comprehensive study that conducts a systematic assessment of the methodological quality and a meta-analysis of current clinical studies on radiomics-based prediction of KRAS status in lung cancer. This meta-analysis represents the first attempt to quantitatively summarize and interpret data from different independent studies, potentially providing critical insights into its clinical applications and future research directions.

A total of 20 studies involving 4,953 patients were included in this meta-analysis. These studies exhibited significant geographical disparities, with 12 studies (60%) originating from China and encompassing 3,736 Chinese patients. This phenomenon aligns with the high incidence of lung cancer in China (39) and the relatively elevated KRAS mutation rate in the Asian region (40).

We further conducted an exploratory bibliometric analysis by searching the PubMed core database. The data revealed that in fields related to “radiomics,” a substantial 43.1% of the papers originated from China; in the realm of “lung cancer” research, Chinese researchers contributed 34.2% of the studies. These figures underscore the emergence of China as a key research force in these two domains. Notably, there has been a significant surge in research output in this field since 2018.

Meanwhile, most radiomics studies demonstrate a high degree of methodological consistency, reflecting the gradual maturation of the field and a positive trend towards standardization (41, 42).

Previous systematic reviews and meta-analyses have confirmed the effectiveness of radiomics in multiple areas, such as predicting the progression and recurrence of hepatocellular carcinoma (43), the occurrence of radiation pneumonitis in lung cancer (44), the overall survival of lung cancer patients (45, 46), the response to neoadjuvant chemotherapy in early-stage breast cancer (47), and lymph node metastasis in esophageal cancer (48). Unlike previous studies, to comprehensively explore the generalization ability of radiomics models, we independently analyzed the results from model training sets and independent validation sets. Compared to the results from the training sets, the validation sets did not show a decline in model performance. Compared to cross-validation methods, using independent validation cohorts can reduce the risk of overfitting, more accurately assess actual performance, achieve a more comprehensive evaluation of model performance, and provide broader generalization capabilities. Independent validation cohorts ensure that models are validated in entirely new datasets, reducing bias and enhancing the accuracy of performance evaluations.

It is noteworthy that the utilization of public datasets for external testing, as employed by Dong et al. (22, 24, 28–30, 34), is an effective strategy for enhancing the generalization ability of radiomics models. Regarding the specific task explored in this meta-analysis, the non-small cell lung cancer (NSCLC) radiogenomics dataset from The Cancer Imaging Archive (TCIA) (49) could serve as a suitable external validation cohort, a point worthy of in-depth exploration in future research.

This systematic review employed the QUADAS-2 (50), RQS (20), and METRICS (42) tools to evaluate the quality of radiomics studies. Overall, the included studies demonstrated a moderate to good quality level. A 2023 review by Tabnak et al. (51) on MRI prediction of Ki-67 expression in breast cancer incorporated 31 studies, with an average RQS of only 5.9 points. In contrast, this systematic review included 20 studies with an average RQS of 9.86 ± 3.7 points, significantly higher than the former, reflecting notable advancements in the field in recent years.

Further analysis of the 16 rating dimensions of the Radiomics Quality Score (RQS) revealed that the “validation” category exhibited the most significant score disparities, while the following five areas scored the lowest: “prospective studies registered in a trial database,” “phantom studies conducted on all scanners,” “multi-time-point imaging,” “biological correlation testing and discussion,” and “cost-effectiveness analysis.” These score differences partly stem from the specific inclusion criteria set in this study. Although prospective study designs are crucial for establishing associations between radiomic features and clinical outcomes in specific patient populations, none of the current included studies adopted such a design framework due to the complex heterogeneity of treatment indications.

The core value of phantom studies lies in validating the consistency of feature extraction across scanning devices from different manufacturers, which is particularly important given that actual evaluation cohorts often involve multi-center collaborations and the use of imaging equipment across different models. Multi-time-point imaging, considering variations in radiomic features due to organ motion or changes in target volume, requires stable image sets obtained through short-term repeated measurement data (two or more image datasets from patients). Biological correlation analysis can reveal the association pathways between imaging phenotypes and underlying molecular mechanisms, providing a critical dimension for understanding disease heterogeneity.

At the level of economic benefit verification, cost-effectiveness analysis necessitates a systematic comparison of the input-output ratios between radiomics techniques and traditional diagnostic and treatment pathways. Its comprehensive application not only relates to the clinical translation value of the model but also provides quantitative evidence for key methodological parameters in subsequent validation stages, including labor costs for image segmentation, resource allocation efficiency for multi-phase scans, and net benefit increments of new technologies relative to existing examination methods. However, accomplishing these tasks in retrospective studies poses significant challenges (52, 53).

Additionally, we evaluated the METRICS scores adopted in the studies. As an emerging evaluation tool, METRICS provides more detailed and balanced evaluation dimensions compared to RQS. For most included studies, there is still room for optimization in handling clinical confounding factors, the rational use of machine learning algorithms, precise selection of imaging stages, independent validation of research results, and ensuring scientific data transparency and reproducibility. We advocate that future radiomics studies should incorporate these key points into their analysis and reporting processes.

Radiomics demonstrates high potential value in predicting the KRAS status in lung cancer. In the training group, the overall sensitivity, specificity, and area under the curve (AUC) were 0.80 (95% CI: 0.76 - 0.83), 0.78 (95% CI: 0.75 - 0.82), and 0.85 (95% CI: 0.82 - 0.88), respectively. The corresponding values in the validation group were 0.79 (95% CI: 0.73 - 0.84), 0.77 (95% CI: 0.71 - 0.82), and 0.85 (95% CI: 0.81 - 0.88). This indicates that radiomics models can effectively distinguish the KRAS status in lung cancer patients. Furthermore, although the Deeks’ test result showed a P-value of 0.71, it is essential to emphasize the limitations of this conclusion. With only 20 included studies and significant regional concentration (60% of the studies originating from China), the insufficient sample size and uneven geographical distribution result in inadequate statistical power for the test, potentially masking potential biases. Future studies should strive to include studies from more regions and expand the sample size of included studies as much as possible to enhance the generalizability and reliability of the results.

The included studies exhibited significant heterogeneity, a result consistent with the conclusions of previous meta-analyses in the field of radiomics (54–57) and in line with the pre-established cognitive framework of this study. Given that all current studies adopted a retrospective research paradigm, the presence of such heterogeneity is methodologically inevitable. Although most studies followed convergent standard operating procedures, multiple methodological decision points exist throughout the analysis chain, including image data preprocessing, feature selection strategies, and model construction algorithms, all of which may influence the results. The cumulative effect of these variables ultimately leads to significant differences among studies, essentially reflecting the inherent methodological vulnerability of the current highly personalized modeling approach.

Nevertheless, the comprehensive statistical results of the current models still hold significant value. Through meta-regression analysis, we delved into the sources of heterogeneity and found that several key methodological factors, including RQS, imaging modality, and modeling algorithms, may be the primary reasons for the heterogeneity among studies. Similar to the study by Spadarella et al. (58), we found that the overall RQS was low and speculated that methodological quality might introduce bias into the pooled results. In the absence of RQS threshold standards, we classified the study cohort based on data-driven hypotheses and found that studies with an RQS ≤ 5 (very low-quality subgroup) had higher predicted specificity, suggesting a possible overestimation. This may be because these studies often failed to perform independent validation or only used internal data for validation while not implementing feature dimensionality reduction, leading to the inclusion of redundant features in the model and overfitting on the training set. This also indicates that different RQS levels can affect research results, confirming an association between RQS and result heterogeneity.

Radiomic features are closely related to the imaging modalities used. Texture features, morphological features, and other features extracted from CT and PET/CT images may not be entirely consistent. Differences in radiomic features generated by different imaging modalities can lead to significant variations in model performance with imaging methods, thereby exacerbating heterogeneity. Compared to traditional radiomic methods, deep learning methods typically have more complex architectures and more parameters. Differences in training strategies, hyperparameter selection, network architectures, and other factors among different studies can also lead to heterogeneity (59).

In summary, we believe that radiomics demonstrates good diagnostic efficacy in predicting the KRAS mutation status in lung cancer. In our subgroup analysis, we found that modeling using PET/CT exhibited superior diagnostic accuracy compared to CT. Previous studies (60, 61) have discovered intriguing associations between PET-derived radiomic features (such as SUVmax and SUVak) and key genetic variations, including KRAS, EGFR, and TP53 mutations. For example, SUVPeak is associated with mutations in the transforming growth factor-β pathway in adenocarcinoma, highlighting the potential of radiogenomics to capture tumor heterogeneity and guide treatment decisions (62). Unlike anatomical imaging techniques that provide static structural information, PET imaging can dynamically reflect metabolic activity, revealing underlying tumor physiological characteristics, which is advantageous for subsequent machine learning analysis. We found that constructing radiomics models using deep learning algorithms can enhance the model’s diagnostic performance. This finding is consistent with previous studies (63–65). Many scholars believe that constructing radiomics models using deep learning algorithms is superior to traditional machine learning algorithms. Deep learning models possess the capability to handle high-dimensional data and complex tasks, and their performance often improves with an increase in data volume. Especially in the case of large datasets, deep learning can fully exploit the value of training data (66).

This study has several limitations. Firstly, all the included studies adopted a retrospective design. Prospective studies are generally considered superior to retrospective ones due to the use of standardized imaging protocols, timely extraction of relevant radiomic features, implementation of standardized blinded data collection, and optimized study designs. Therefore, caution is warranted when directly applying these research findings, as there may be concerns regarding generalizability. Secondly, during the data extraction process, if a study presented multiple models, we selected the one with the best diagnostic performance. This approach may have overestimated the sensitivity and specificity of radiomics models in distinguishing the KRAS status in lung cancer. Thirdly, the codes used for feature extraction and model construction in the studies were not publicly available, which hinders the validation of the research results. Finally, not all included datasets met the ideal sample size criterion (i.e., a sample size ten times the number of model features), highlighting the necessity for future studies to establish larger-scale clinical cohorts or reduce the number of model features.

## Conclusion

In conclusion, this meta-analysis indicates that radiomics is a promising tool for determining the KRAS mutation status in lung cancer. However, the quality of radiomics research still needs improvement. Strictly adhering to relevant guidelines for radiomics research is crucial. Additionally, prospective clinical trials should be designed to validate the practical applicability of the models and promote their clinical application.

## Data Availability

The original contributions presented in the study are included in the article/**Supplementary Material**. Further inquiries can be directed to the corresponding authors.
