# Supplementary material for: Quality and accuracy of radiomics models in predicting KRAS status in lung cancer: a systematic review and meta-analysis
Source: Front Oncol. 2026 Jan 9;15:1701122. doi: 10.3389/fonc.2025.1701122 (PMC12827150; doi:10.3389/fonc.2025.1701122)
Supplement: Supplementary file 1 [file DataSheet1.pdf]

Supplementary Material

Quality and Accuracy of Radiomics Models in Predicting KRAS Status in Lung Cancer: A Systematic Review and Meta-Analysis

Table S1. PubMed searching algorithm

Set	Query
#1	Lung Neoplasms[MeSH Terms]
#2	((((((((((((((((Pulmonary Neoplasms) OR (Neoplasms, Lung)) OR (Lung Neoplasm)) OR (Neoplasm, Lung)) OR (Neoplasms, Pulmonary)) OR (Pulmonary Neoplasm)) OR (Lung Cancer)) OR (Cancer, Lung)) OR (Cancers, Lung)) OR (non-small cell lung cancer)) OR (Carcinoma, Non-Small-Cell Lung)) OR (Lung Adenocarcinoma)) OR (Lung Adenocarcinomas)) OR (Lung Adenocarcinoma)) OR (Adenocarcinoma, Lung)) OR (Lung Squamous Cell Carcinoma)) OR (Squamous Cell Carcinoma of the Lung)) OR (Pulmonary Squamous Cell Carcinoma)
#3	#1 OR #2
#4	kirsten rat sarcoma[MeSH Terms]
#5	((kirsten rat sarcoma) OR (KRAS)) OR (RAS)
#6	#4OR#5
#7	Radiomics[MeSH Terms]

**#8**	**((((((((((Radiomics) OR (Texture Analysis)) OR (Image Features)) OR (Computer-Aided Diagnosis)) OR (Quantitative Imaging)) OR (artificial Intelligence)) OR (machine intelligence)) OR (computer vision systems)) OR (machine learning)) OR (deep learning)) OR (supervised machine learning)) OR ("support vector machine)**
**#9**	**#7OR#8**
**#10**	**#3AND#7AND#9**

Table S2. Embase searching algorithm

**Set**	**Query**
**#1**	**'lung cancer'/exp**
**#2**	**‘Pulmonary Neoplasms’OR‘Neoplasms, Lung’OR‘Lung Neoplasm’OR‘Neoplasm, Lung’OR‘Neoplasms, Pulmonary’OR‘Pulmonary Neoplasm’OR ‘Lung Cancer’OR‘Cancer, Lung’OR‘Cancers, Lung’OR‘non-small cell lung cancer’OR‘Carcinoma, Non-Small-Cell Lung’OR‘Lung Adenocarcinoma’OR‘Lung Adenocarcinomas’OR‘Lung Adenocarcinoma’OR‘Adenocarcinoma, Lung’OR‘Lung Squamous Cell Carcinoma’OR‘Squamous Cell Carcinoma of the Lung’OR‘Pulmonary Squamous Cell Carcinoma’**
**#3**	**#1 OR #2**
**#4**	**‘kirsten rat sarcoma’/exp**
**#5**	**‘kirsten rat sarcoma’OR‘KRAS’OR‘RAS’**

**#6**	**#4 OR #5**
**#7**	**'Radiomics'**
**#8**	**'Radiomics'OR'Texture Analysis'OR'Image Features'OR'Computer-Aided Diagnosis'OR'Quantitative Imaging'OR'artificial Intelligence'OR'machine intelligence'OR'computer vision systems'OR'machine learning'OR'deep learning'OR'supervised machine learning'OR'support vector machine'**
**#9**	**#7 OR #8**
**#10**	**#3AND#7AND#9**

Table S3. Web of Science searching algorithm

**Set**	**Query**
**#1**	**TS=(Lung Neoplasm* OR Pulmonary Neoplasms*, Lung OR Neoplasm*, Pulmonary OR Pulmonary Neoplasm*OR Lung Cancer* OR Cancer, Lung* OR Pulmonary Cancer* OR Cancer*, Pulmonary OR Cancer* of the Lung OR Cancer* of Lung OR Lung Adenocarcinoma* OR Adenocarcinoma*, Lung OR Adenocarcinoma of Lung，Lung Squamous Cell Carcinoma*，Squamous Cell Carcinoma of the Lung*，Pulmonary Squamous Cell Carcinoma*)**
**#2**	**TS=（kirsten rat sarcoma OR KRAS OR RAS）**
**#3**	**TS=（Radiomics OR Texture Analysis OR Image Features OR Computer-Aided Diagnosis OR Quantitative Imaging*，artificial Intelligence*，machine intelligence*，computer vision systems OR machine learning OR deep learning*，supervised machine learning OR support vector machine*）**

**#4**	**#3 AND #2 AND #1**

Table S4. Cochrane searching algorithm

**Set**	**Query**
**#1**	**MeSH descriptor: [Lung Neoplasms] explode all trees**
**#2**	**MeSH descriptor: [Adenocarcinoma of Lung] explode all trees**
**#3**	**(Lung Neoplasm* OR Neoplasm*, Lung OR Neoplasm*, Pulmonary OR Pulmonary Neoplasm* OR Lung Cancer* OR Cancer, Lung* OR Pulmonary Cancer* OR Cancer*, Pulmonary OR Cancer* of the Lung OR Cancer* of Lung OR Lung Adenocarcinoma* OR Adenocarcinoma*, Lung OR Adenocarcinoma of Lung， Lung Squamous Cell Carcinoma*， Squamous Cell Carcinoma of the Lung*， Pulmonary Squamous Cell Carcinoma*):ti,ab,kw**
**#4**	**#1 OR #2 OR #3**
**#5**	**MeSH descriptor: [kirsten rat sarcoma] explode all trees**
**#6**	**（kirsten rat sarcoma，KRAS，RAS）**
**#7**	**#5 OR #6**
**#8**	**MeSH descriptor:[Radiomics]explode all trees**

#9	（Radiomics OR Texture Analysis OR Image Features OR Computer-Aided Diagnosis OR Quantitative Imaging*，artificial Intelligence*，machine intelligence*，computer vision systems OR machine learning OR deep learning*，supervised machine learning OR support vector machine*）:ti,ab,kw
#10	#8 OR #9
#11	#4 AND # 7 AND #10

Table S5. China National Knowledge Infrastructure searching algorithm

	Title	Lung cancer + non-small cell lung cancer + bronchogenic carcinoma + primary bronchogenic carcinoma + bronchogenic carcinoma + NSCLC+ Pulmonary Squamous Cell Carcinoma
AND	Title	kirsten rat sarcoma+KRAS+RAS
AND	Title	Radiomics+ Texture Analysis+ Image Features+ Quantitative Imaging+ Computer-Aided Diagnosis+ artificial Intelligence+ machine learning

Table S6. Preferred Reporting Items for a Systematic Review and Meta-analysis of Diagnostic Test Accuracy Studies (PRISMA-DTA) Checklist

Section and Topic	Item No.	PRISMA-DTA Checklist Item
**Title**		

Title	1	Identify the report as a systematic review (meta-analysis) of DTA studies.
Abstract	2	replaced this item with the PRISMA-DTA checklist for abstracts.
**Introduction**		
Rationale	3	Describe the rationale for the review in the context of what is already known
Clinical role of index test	D1	State the scientific and clinical background, including the intended use and clinical role of the index test, and if applicable, the rationale for minimally acceptable test accuracy (or minimum difference in accuracy for a comparative design)
Objectives	4	Provide an explicit statement of question(s) being addressed in terms of participants, index test(s), and target condition(s).
**Methods**		
Protocol and registration	5	Indicate where the review protocol can be accessed (eg, web address) and provide trial registration number if available.
Eligibility criteria	6	Specify study characteristics (participants, setting, index test, reference standards, target conditions, and study design) and report characteristics (eg, years considered, language, publication status) used as criteria for eligibility and providing rationale.
Information sources	7	Describe all information sources (eg, databases with dates of coverage, contact with study authors to identify additional studies) in the search and the date last searched.
Search	8	Present full search strategies for all electronic databases and other sources searched, including any limits used so that

		they can be repeated.
Study selection	9	State the process for selecting studies (ie, screening, eligibility, whether included in systematic review, and, if applicable, included in the meta-analysis).
Data collection process	10	Describe the methods of data extraction from reports (eg, piloted forms, independently, in duplicate) and any processes for obtaining and confirming data from the investigators.
Definitions for data extraction	11	Provide definitions used in data extraction and classifications of target conditions, index tests, reference standards, and other characteristics (eg, study design, clinical setting).
Risk of bias and applicability	12	Describe methods used for assessing risk of bias in individual studies and concerns regarding the applicability to the review question.
Diagnostic accuracy measures	13	State the principal diagnostic accuracy measures reported (eg, sensitivity, specificity) and state the unit of assessment (eg, per patient vs per lesion).
Synthesis of results	14	Describe the methods of handling the data, combining the results of the studies and describing the variability between studies. This could include, but is not limited to (1) handling of multiple definitions of the target condition, (2) handling of multiple thresholds of test positivity, (3) handling multiple index test readers, (4) handling of indeterminate test results, (5) grouping and comparing tests, and (6) handling of different reference standards.
Meta-analysis	D2	Report the statistical methods used for meta-analyses if performed.
Additional analyses	16	Describe the methods of the additional analyses (eg, sensitivity or subgroup analyses, meta-regression) if done, indicating which were prespecified.

**Results**		
Study selection	17	Provide the numbers of studies screened, assessed for eligibility, included in the review, and included in the meta-analysis if applicable, with reasons for exclusions at each stage, ideally with a flow diagram.
Study characteristics	18	For each included study, provide citations and present key characteristics including (1) participant characteristics (presentation, prior testing), (2) clinical setting, (3) study design, (4) target condition definition, (5) index test, (6) reference standard, (7) sample size, and (8) funding sources.
Risk of bias and applicability	19	Present evaluation of risk of bias and concerns regarding applicability for each study.
Results of individual studies	20	For each analysis in each study (eg, unique combination of index test, reference standard, and positivity threshold), report 2 × 2 data (TP, FP, FN, TN) with estimates of diagnostic accuracy and confidence intervals, ideally with a forest plot or a receiver operating characteristic curve.
Synthesis of results	21	Describe test accuracy, including variability; if meta-analysis was done, include results and confidence intervals.
Additional analyses	23	Give results of additional analyses if done (eg, sensitivity or subgroup analyses, meta-regression, analysis of index test, failure rates, proportion of inconclusive results, and adverse events).
**Discussion**		
Summary	24	Summarize the main findings including the strength of the evidence.
Limitations	25	Discuss limitations from included studies (eg, risk of bias and concerns regarding applicability) and from the review

		process (eg, incomplete retrieval of identified research).
Conclusions	26	Provide a general interpretation of the results in the context of other evidence. Discuss implications for future research and clinical practice (eg, the intended use and clinical role of the index test).
**Other**		
Funding	27	For the systematic review, describe the sources of funding and other support and the role of the funders.

Table S7 Results of the 2×2 contingency table

Study ID	TP（T/V）	FP（T/V）	FN（T/V）	TN（T/V）	Sensitivity %（T/V）	Specificity %（T/V）
Rizzo（2014）（19）	48.00	60.00	16.00	161.00	0.75	0.73
Velazquez (2018)(20)	172.00	148.00	43.00	400.00	0.80	0.72
Rizzo(2019)(21)	34.00	20.00	10.00	58.00	0.78	0.36
Dong (2020)(22)	60.00/21	72.00/26	24.00/9	207.00/68	0.71/0.69	0.74/0.73
Moreno（2021）（23）	17.00	16.00	8.00	58.00	0.66	0.78
Nguyen(2021)(24)	19.00/1	10.00/1	8.00/2	77.00 /14	0.71 /0.33	0.89 /0.93
Zhang (2021)（25）	13.00	38.00	2.00	81.00	0.87	0.68
Shiri (2022)(26)	14.00 /10	8.00 /9	5.00 /2	43.00 /44	0.73 /0.81	0.84 /0.83

Wang (2022)(27)	86.00/37	17.00 /7	4.00 /2	73.00 /32	0.96 /0.95	0.81 /0.87
Chen (2023)（28）	48/26	39/21	11/8	148/69	0.81/0.77	0.79/0.77
Prencipe（2023）（29）	11.00 /12	8.00 /9	3.00 /3	38.00 /31	0.81 /0.84	0.83 /0.78
Zhang (2023)(30)	21.00 /2	14.00 /1	9.00 /1	52.00 /2	0.67 /0.67	0.79 /0.67
Kohan（2024）（31）	34.00	34.00	7.00	82.00	0.83	0.71
Li(2024)(32)	18.00	10.00	4.00	48.00	0.81	0.82
Lv（2024）（33）	31.00/16	27.00/15	7.00 /2	153.00 /66	0.83/0.9	0.85/0.81
Xue(2024)(34)	21.00/5	10.00/3	3.00/1	65.00/16	0.86/0.82	0.86/0.83
Xu（2024）（35）	31.00 /10	35.00 /10	9.00 /6	94.00 /47	0.77 /0.64	0.73 /0.83
Fu （2025）（36）	22.00	6.00	9.00	32.00	0.71	0.92
Mahmoud（2025）（37）	68.00 /66	117.00 /212	17.00 /19	613.00 /518	0.81 /0.78	0.84 /0.71
Schöneck（2025）（38）	25/13	45/22	15/5	63/24	0.62/0.72	0.58/0.52

Abbreviations: T：Training；V：Validation。
